# Protocol to combine brain sections from multiple mice into a single block for spatial transcriptomic analyses

**DOI:** 10.1016/j.xpro.2023.102617

**Published:** 2023-09-23

**Authors:** Yoshitaka J. Sei, Myriam M. Chaumeil, Ken Nakamura

**Affiliations:** 1 Gladstone Institute of Neurological Disease, Gladstone Institutes, San Francisco, CA 94158, USA; 2 Department of Physical Therapy and Rehabilitation Science, San Francisco, CA 94158, USA; 3 Department of Radiology and Biomedical Imaging, San Francisco, CA 94158, USA; 4 UCSF/UCB Graduate Program in Bioengineering, University of California San Francisco, San Francisco, CA 94158, USA; 5 Graduate Program in Biomedical Sciences, University of California San Francisco, San Francisco, CA 94143, USA; 6 Graduate Program in Neuroscience, University of California San Francisco, San Francisco, CA 94158, USA; 7 Department of Neurology, University of California, San Francisco, San Francisco, CA 94158, USA

**Keywords:** Genomics, Neuroscience, Biotechnology and Bioengineering

## Abstract

Spatial transcriptomics couples visual spatial markers with gene expression levels, but slide space and cost limit the number of samples that can be processed. Here, we present a protocol for mounting brains from multiple mice onto a single capture area of a spatial transcriptomics slide. We describe steps for conjoining frozen hippocampal sections from different brains into a single cryostat block, reducing the quantity of reagents required. This protocol is applicable to a range of existing spatial genomics platforms.

For complete details on the use and execution of this protocol, please refer to Li et al. (2023).^1^

## Highlights

• Protocol to assay multiple brains in each spatial transcriptomics capture area
• Merge frozen tissues from multiple mouse brains into a single cryostat block
• Trim samples to isolate regions of interest
• Approach conserves resources and improves statistical power and cost efficiency

## Abstract (editor highlights)

Publisher’s note: Undertaking any experimental protocol requires adherence to local institutional guidelines for laboratory safety and ethics.

## Graphical abstract

**Figure:**
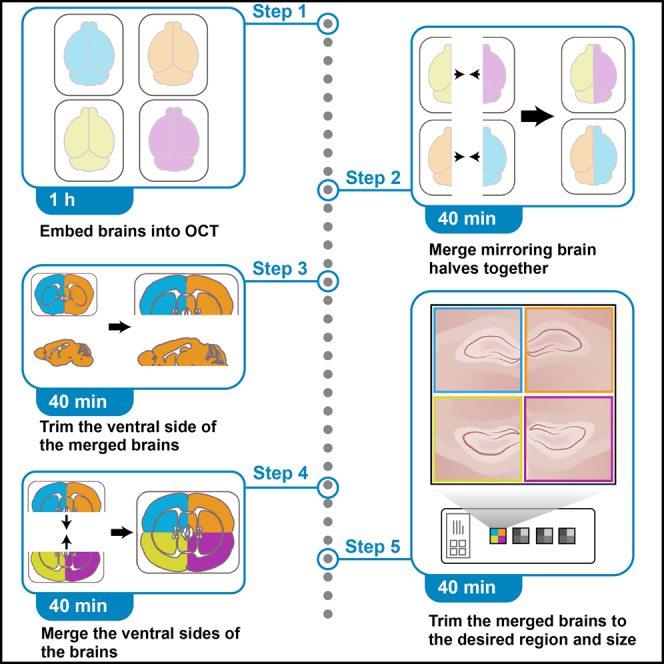

## Before you begin

The protocol below describes specific steps for preparation of fresh-frozen mouse brains for use with spatial transcriptomic platforms. It is critical that institutional approval is obtained prior to work with live animals. This protocol uses the hippocampus as its region of focus. This protocol requires the use of a cryostat.

### Institutional permissions

All procedures were approved by the Institutional Animal Care and Use Committee at the Gladstone Institutes and University of California San Francisco in accordance with the National Institutes of Health guidelines.

### Clean and set temperature of cryostat

**Timing: 30 min**

1. For brain tissue, set the cryostat specimen temperature to -12C and the chamber temperature to −18°C.
2. Wipe down the inside of the cryostat with 70% ethanol sprayed onto a paper towel.
3. Spray RNAseZap onto the paper towel and clean the specimen holders and the section collection stage.
  a. Be careful not to leave the paper towel with RNAseZap on the freezing surface as it will cause the paper towel to stick.
4. Clean the sectioning blade with RNAseZap prior to installing into the cryostat.
5. Clean brushes, tweezers, and razers with RNAseZap prior to cooling them in the cryostat.

### Embed fresh-frozen brains into OCT

**Timing: 1 h**

6. Dispense OCT into cryomolds on ice.
7. Chill OCT on ice for at least 30 min.
8. Have powdered dry ice ready to freeze OCT-embedded brains.
  a. Alternatively, prepare a slurry of dry ice and isopropanol for freezing samples.
9. Retrieve fresh-frozen brains from storage.
10. With ice-cold tweezers, quickly place a frozen brain at the bottom of an OCT-filled mold.
11. Use the tweezers to quickly spread a layer of OCT over the frozen brain.
12. Dispense a thicker layer of ice-cold OCT on top of the brain.
  a. The surface of the brain may appear to show signs of thawing at this stage.
13. Quickly place the OCT-embedded brains onto the powdered dry ice (or another surface at −80°C) and allow OCT to freeze completely.
14. Store embedded samples at −80°C if the cryostat will be used on a separate day.

## Key resources table

**Table:** 

REAGENT or RESOURCE	SOURCE	IDENTIFIER
**Antibodies**		

Rabbit monoclonal anti-NeuN (1:200)	Abcam	Cat#ab177487; RRID: AB_2532109
Mouse monoclonal Anti-Glial Fibrillary Acidic Protein Antibody, clone GA5 (1:100)	Sigma-Aldrich	Cat#MAB3402; RRID:AB_94844
Hoechst (final working concentration: 1 μg/mL)	Invitrogen	Cat#H1399

**Chemicals, peptides, and recombinant proteins**		

RNAseZAP	Ambion	Cat#AM9780
KOPTEC Ethanol 200 Proof (for 70% ethanol)	VWR	Cat#89125-188
Tissue Tek OCT Compound	VWR	Cat#25608-930

**Critical commercial assays**		

Spatial gene expression slide: Example – Visium Spatial Gene Expression Kit	10× Genomics	CAT#1000184

**Experimental models: Organisms/strains**		

Mouse: GLUT3^lox/lox^ (7 months)	Contat et al.^2^	
Mouse: B6.Cg-Tg(Camk2a-cre)T29-1Stl/J (7 months)	Jackson Laboratories	JAX: 005359

**Software and algorithms**		

Allen Institute for Brain Science: Allen Brain Explorer	Oh et al.^3^	http://connectivity.brain-map.org/3d-viewer?v=1
	Lein et al.^4^	
	Harris et al.^5^	
	Daigle et al.^6^	

**Other**		

American Safety Razors Single Edge Blade	Fisher Scientific	CAT#S04615
Standard pattern forceps	Fine Science Tools	CAT#11000-12
CM1900 Cryostat	Leica	N/A
Disposable microtome blades	Leica	CAT#63065-HP
Andwin Scientific Cryomold	Fisher Scientific	CAT#NC9511236
Stainless steel spatula	Fisher Scientific	CAT#13-820-056
Permanent laboratory markers	VWR	CAT#52877
Ethanol	VWR	CAT#89125
Sectioning brushes	Ted Pella	CAT#11860
Dry ice		
Ice		

## Step-by-step method details

### Assembly of brain blocks for hippocampal sections

**Timing: 2 h**

This step allows the assembly of brains from four mice into a single OCT block for spatial transcriptomics of hippocampal sections.

1. Clean gloved hands with RNAseZap.
2. Use colored permanent sample markers to assign different colors to the OCT of 4 brain samples (Figure 1A).

**Figure 1:**
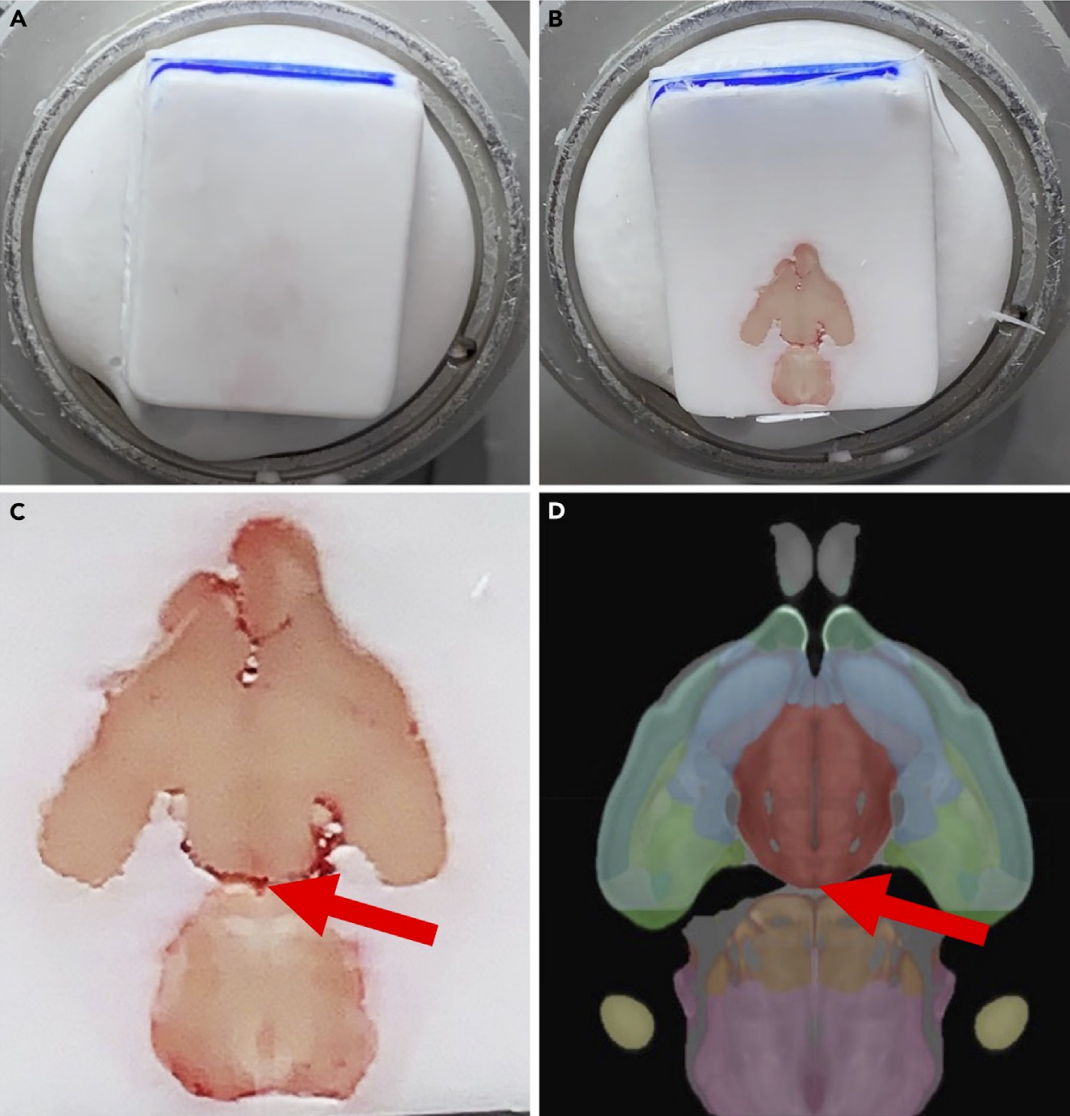
Identifying optimal trimming depth in mouse brains (A) Mount OCT-embedded brains in the cryostat to trim from ventral side of the brain. A colored laboratory pen is used to label the OCT block. Different colors should be used for different brains. (B) View of brain when sufficient trimming is achieved. (C) Optimal trimming depth is reached when the brainstem appears to come in contact with the hypothalamus. The midbrain flexure will be apparent (red arrow) between the hypothalamus and brainstem. (D) Allen Brain Atlas view of optimal trimming depth with focus on brainstem coming together with hypothalamus (red arrow indicates location of expected midbrain flexure).^3^^,^^4^^,^^5^^,^^6^
3. Position the first sample in the cryostat so that the ventral surface of the brain is the face being cut.
4. Trim brain 1 from the ventral side to such depth that the brainstem and the hypothalamus appear to touch (separated by the midbrain flexure) in the same plane (Figures 1B–1D).
5. Use a single-edge razor to cut the brain in half down the sagittal plane (Figure 2).

**Figure 2:**
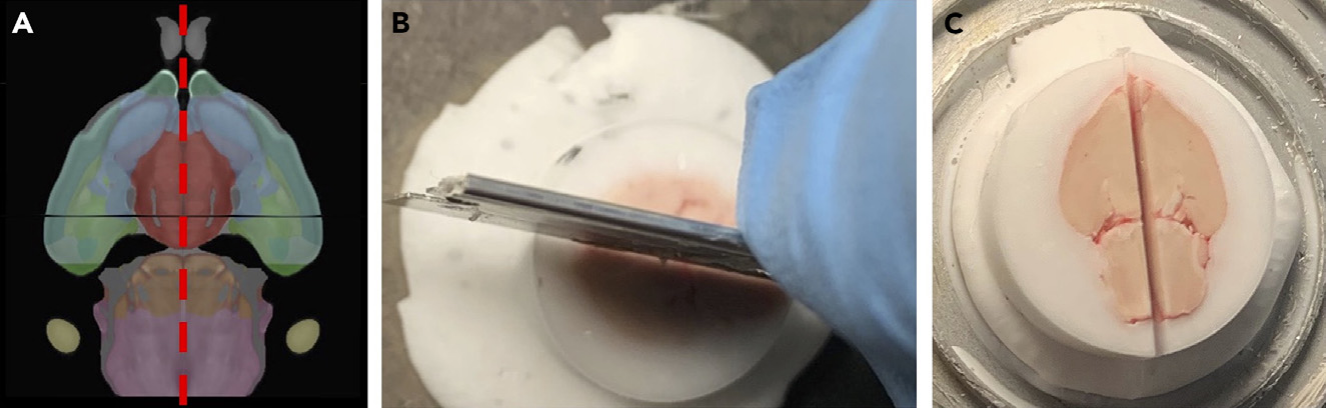
Dividing the brain into right and left hemispheres (A) Allen Brain Atlas view of the path of the incision through the midline of the brain (red-dotted line). (B) Use a razer chilled in the cryostat to cut the brain in half (make the cut as straight as possible). (C) The resulting cut should be right through the middle of the brain.^3^^,^^4^^,^^5^^,^^6^
6. Remove the 2 halves of brain 1 from the mounting block (use a spatula or tweezers).
7. Complete steps 1–5 for brains 2, 3, and 4.
8. Select one half of brain 1 and a mirroring half from brain 2 that you want to merge together.
9. Mount each selected brain half from Step 7 to a separate cryostat chuck with OCT, ensuring the midsagittal plane is face up (with a single-edge razor, trim any excess OCT that obstructs mounting the brain half in this orientation). This enables the user to handle each brain half by holding onto the chuck, rather than the sample itself.
10. Practice aligning the brain halves by matching the midbrain flexure of each brain (Figure 3). Note that samples warm quickly when handled in the cryostat, so the user must align the brains quickly during the merging process.

**Figure 3:**
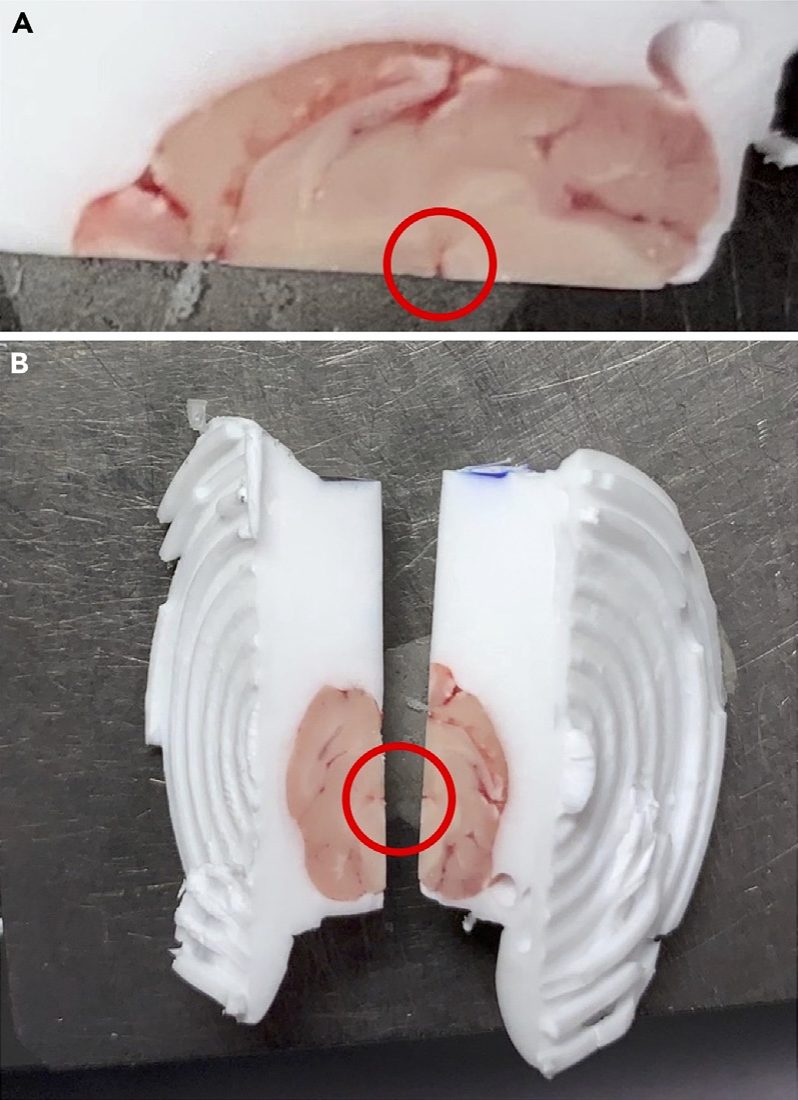
Alignment of 2 brain halves (A) The landmark to align the brain halves to each other is the midbrain flexure (circled in red). (B) Practice aligning the brains together by joining the 2 brains together at the brainstem (red circle). Do this before applying OCT and attempting to join the 2 halves.
11. Gently warm up the sagittal face of each sample to be merged. The user can do this by tapping a room-temperature spatula (treated with 70% ethanol and RNaseZap) on the sagittal face of the brain half just until the OCT starts to soften (the brain should remain frozen).
12. Apply a thin layer of OCT to the sagittal face of the brain 1 half. Firmly press the sagittal face of the brain 2 half into the OCT on the brain 1 half, making sure to align the midbrain flexure.
13. Allow the OCT of the merged halves for brains 1 and 2 to refreeze in the cryostat. Remove the brains from each cryostat chuck one chuck at a time (Figure 4).

**Figure 4:**
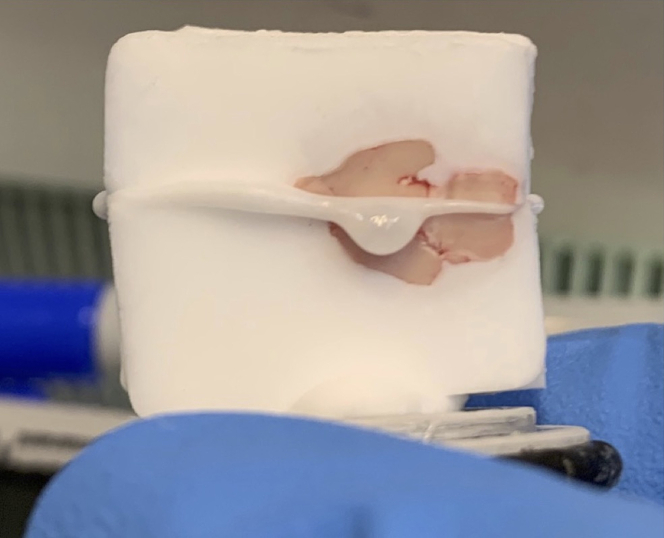
Merging of 2 brain halves with OCT The thin layer of OCT should be applied to the sagittal face of the brains. Spillover along the full length of the brain indicates proper distribution of OCT. Remove the cryostat chucks from these merged halves one at a time.
14. Merge the remaining halves for brains 3 and 4 using Steps 9–13.
15. Remount the merged halves for brains 1 and 2 on the cryostat so that the ventral surface is the face being cut.
16. Shave the bottom face of the merged brains until 2 sets of ventricles appear (Figure 5).

**Figure 5:**
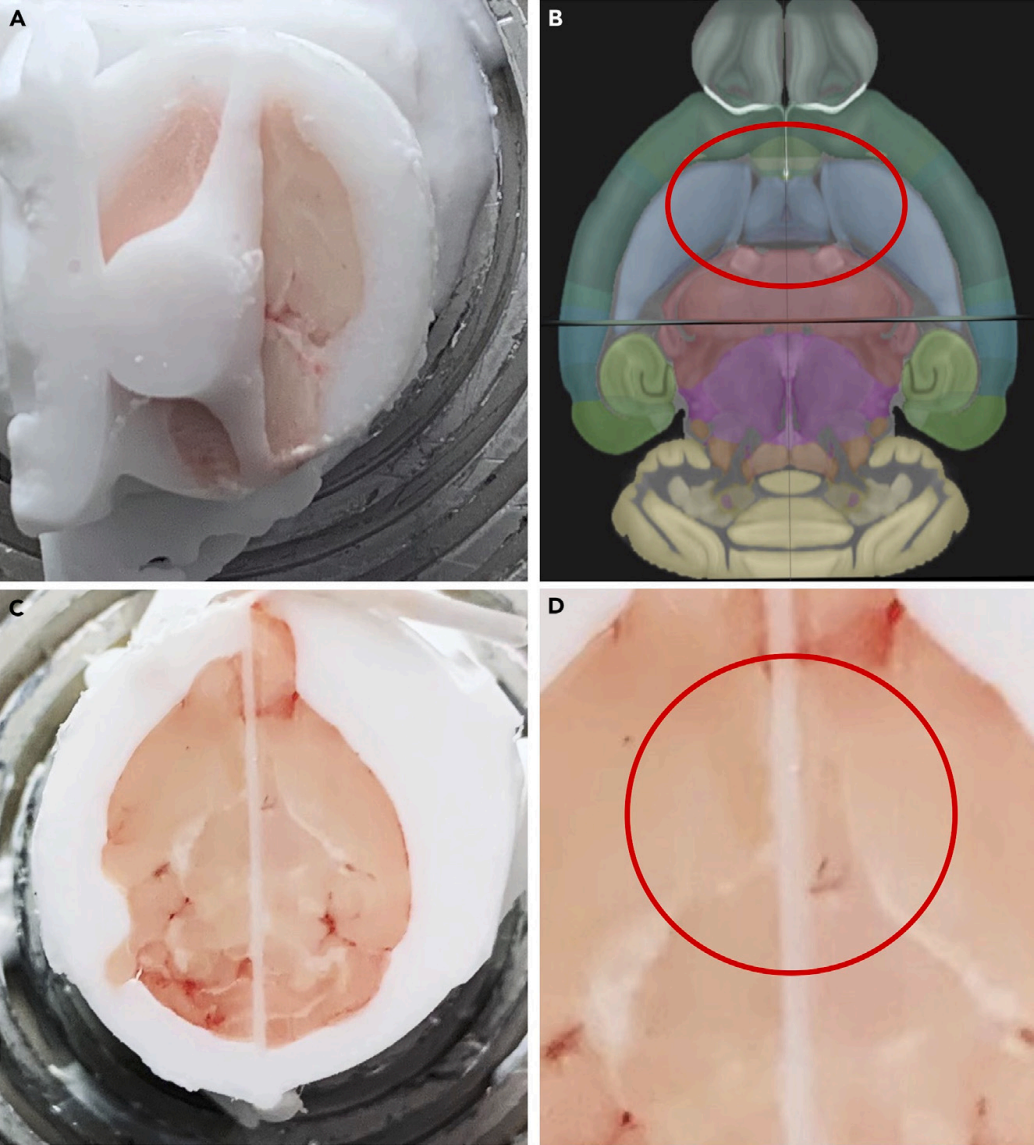
Trimming merged brain halves (A) Merged brain halves should be mounted such that OCT spillover is trimmed across the long side. (B) Allen Brain Atlas view of ventricle landmarks to look for in the horizontal plane (red circle).^3^^,^^4^^,^^5^^,^^6^ (C) Ventricles will appear as white streaks that can sometimes be faint to see. (D) Closer examination of trimmed brain halves reveals white streaks of ventricles (red circle).
17. When the desired ventricle landmark appears, make a horizontal incision with a single-edge razor through both brains, where the thalamus and striatum appear to meet (the red-dotted line) in the plane (Figure 6). Discard rostral part of the block.

**Figure 6:**
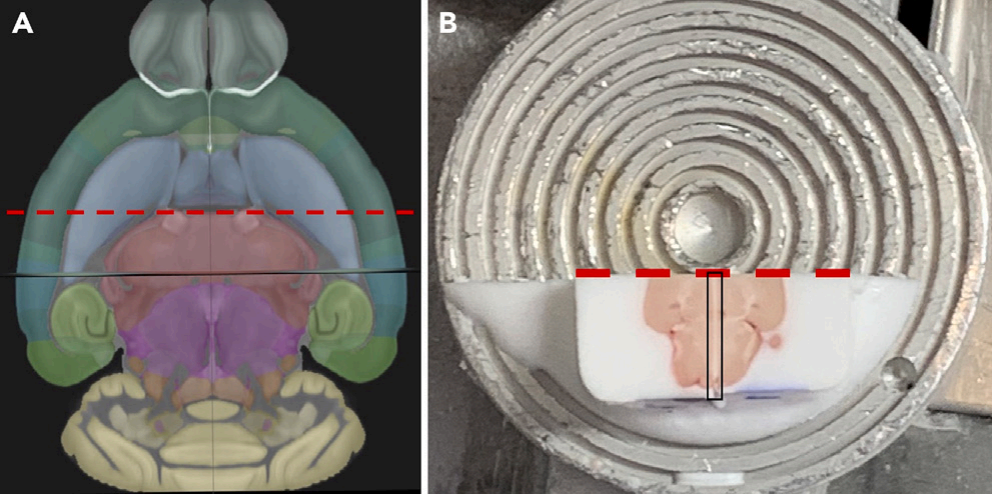
Trimming merged halves for alignment with another pair of merged halves (A) Allen Brain Atlas horizontal plane view of the coronal cut through the striatum (red dotted line) made to align 2 pairs of merged brain halves.^3^^,^^4^^,^^5^^,^^6^ (B) The cut (dotted red line) should be perpendicular to the OCT visible between the brain halves (black outline).
18. The coronal view of the merged brain halves should show the striatum with ventricles appearing as dots (Figure 7).

**Figure 7:**
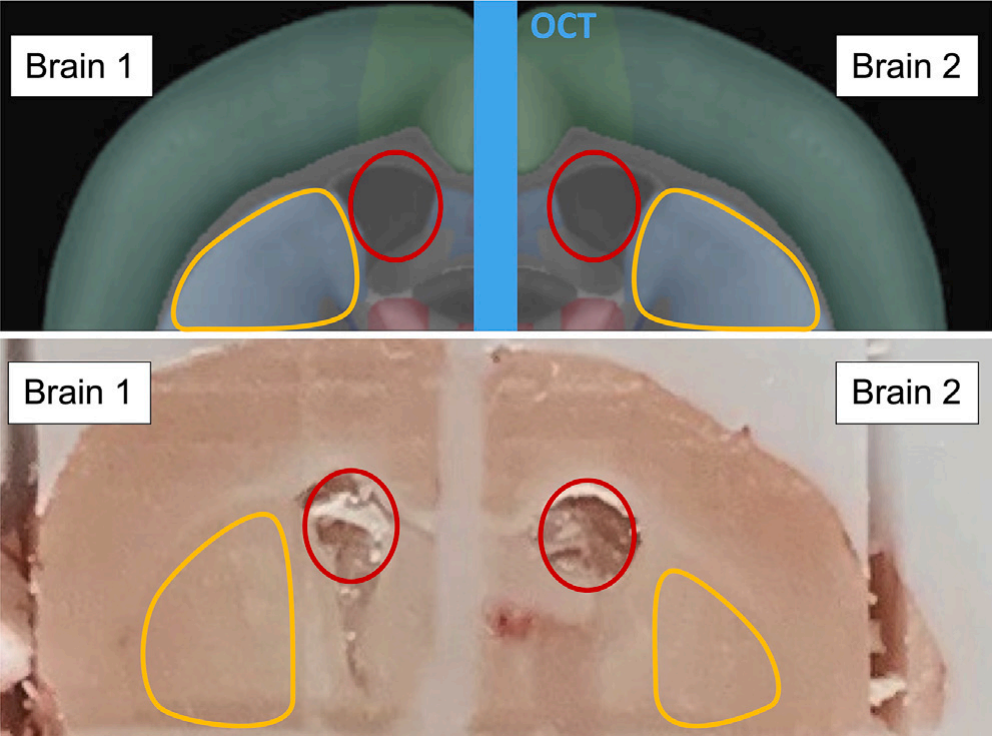
Coronal view of trimmed brain halves Ventricle landmarks (red circles) should be visible above the striatum (yellow outlines) in each brain after the coronal trim. Top, Allen Brain Atlas view.^3^^,^^4^^,^^5^^,^^6^
19. Repeat steps 12–15 to get the merged block of samples 3 and 4 trimmed to the same landmarks.
20. Merge the sample 1–2 block with the sample 3–4 block at the horizontal plane using the same technique of briefly warming up the samples and applying OCT in a thin layer.
  a. Limit handling of the samples by holding onto the mounting block, to minimize warming the tissue.
21. Once fully merged, the coronal view of the untrimmed brain block should show each sample appearing in a separate quadrant (Figure 8).

**Figure 8:**
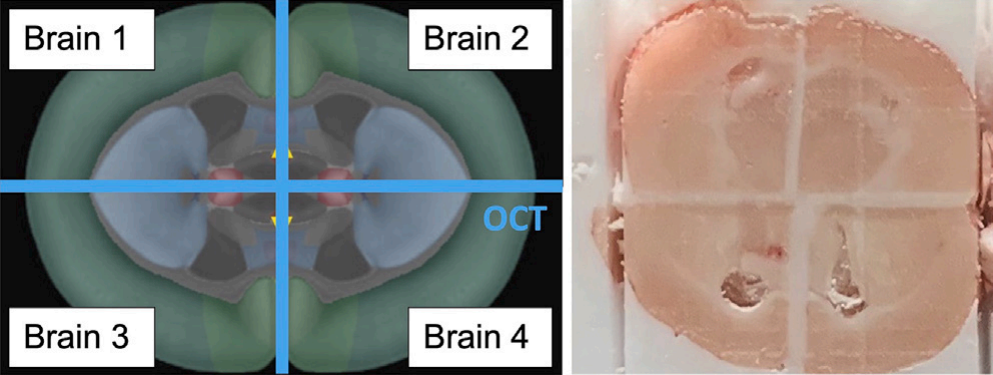
Coronal view of fully assembled brain block Aligned at the coronal cut, 2 pairs of merged brain halves assembled together with OCT. Left, Allen Brain Atlas view.^3^^,^^4^^,^^5^^,^^6^
22. To prepare blocks for mounting, trim the coronal face of the brains until the hippocampus is visible in all 4 brains (Figure 9A).

**Figure 9:**
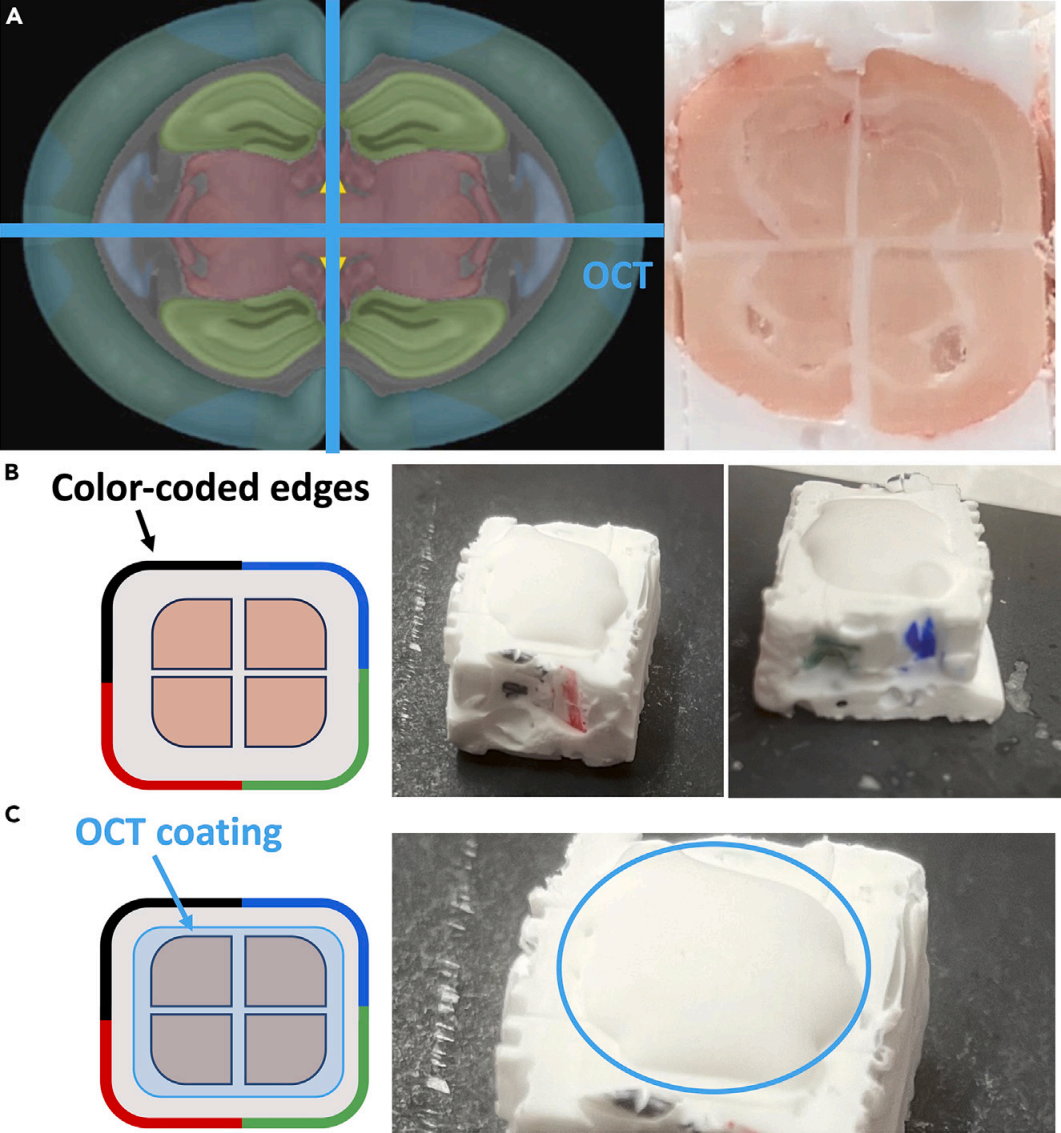
Views of the trimmed brain block (A) Hippocampus will appear as the ventricle landmarks move away from the OCT midline. Left, Allen Brain Atlas view.^3^^,^^4^^,^^5^^,^^6^ (B) Ensure that the color coding from Step 2 is still legible. Recolor the sides of the assembled brain block if necessary. (C) Reapply a thin coat of OCT on the exposed brain area if storing them at -80C in a sealed container for future processing.
23. Ensure that the color-coding from Step 2 is still visible on the sides of the OCT block. Recolor the sides if starting to fade (Figure 9B).
24. If storing the assembled brain block for future processing, apply a thin coat of OCT on exposed brain areas. Allow the OCT to set before putting the brain block into a sealed container at −80°C (Figure 9C).
  a. A 50 mL conical tube works well as a storage container for the brain blocks

## Expected outcomes

**Figure 10:**
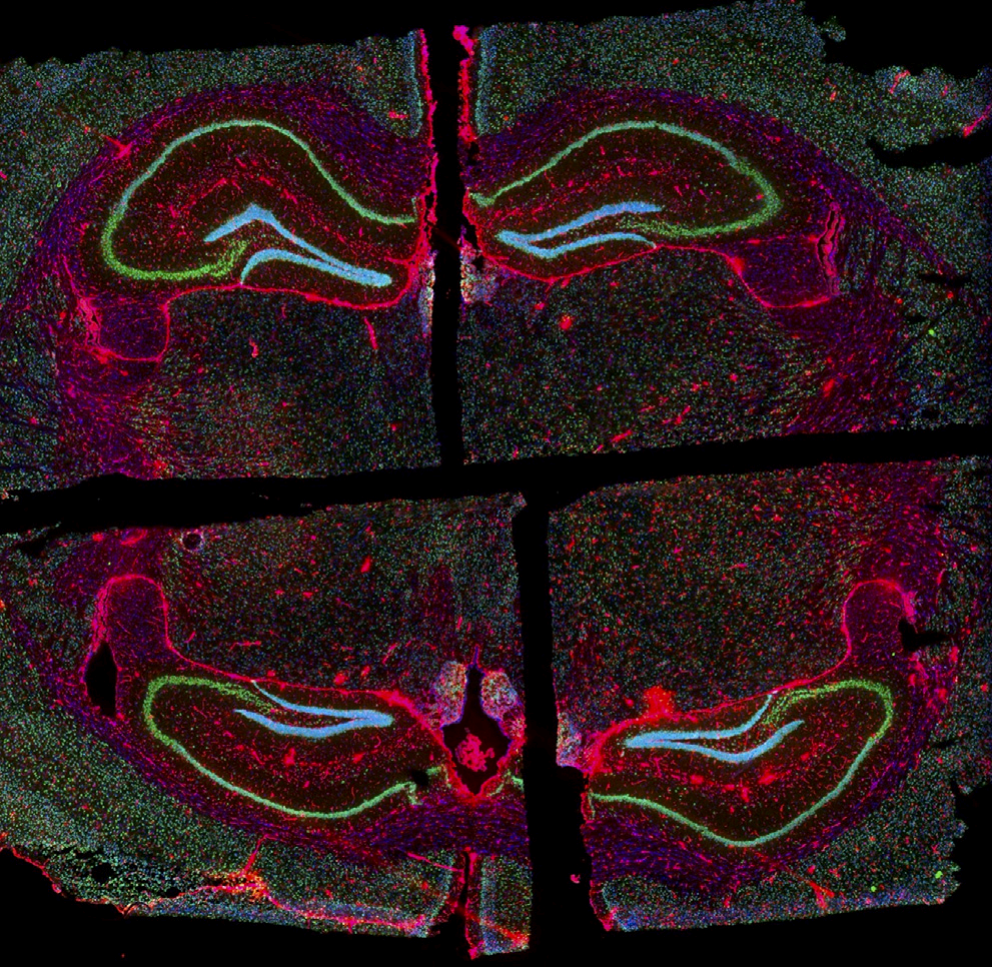
Expected results from trimmed brain block Brains from 4 separate mice were made into a single brain block for a spatial transcriptomic study described in Li et al.^1^ The brain block was trimmed to the hippocampus and stained for neurons (NeuN, green), astrocytes (Gfap, red), and cell nuclei (Hoechst, blue). These floxed GLUT3 mice crossed with CamKII⍺ (CamKcre) mice were used to study the reliance of neurons on glucose uptake and glycolysis.^2^

The final sections to be used for spatial transcriptomics should show 4 quadrants from different brains at similar depths that fit within the borders of the spatial transcriptomic capture area. An example section from Li et al.^1^ shows hippocampi from 4 mice stained for neurons (NeuN), astrocytes (Gfap), and cell nuclei with Hoechst (Figure 10).

## Limitations

The cryostat temperatures listed here are for cryostats that have separate settings for the chamber and the head. Different temperatures may be needed for cryostats that have only a single temperature setting.

## Troubleshooting

### Problem 1: Brains are distorted during the freezing process

If not placed carefully in a dip tray or spatula, brains can retain bends and distortions during flash freezing. Distorted brains are difficult to align during the block assembly process.

### Potential solution

In a container filled with dry ice, chill a bath of isopentane for ∼30 min. Gently place a freshly dissected brain onto a plastic weigh-boat or plastic dip tray to ensure the brain is straight and not damaged during freezing. Submerge the brain into the chilled isopentane bath for a few seconds until the brain is frozen. Move the brain into a dry plastic container chilled to -80C.

### Problem 2: Brains are embedded into OCT at an angle

If the ventral side of the brain does not lie flat when embedded in OCT, it can be difficult to make cuts that are level with the horizontal plane of the brain, which is important when trimming on the cryostat.

### Potential solution

Use tweezers to quickly set the brain into the OCT mold. Then carefully and very quickly apply a thin coat of OCT over the brain before topping off the mold with OCT.

### Problem 3: Poor alignment when joining brains together with OCT

Misalignment during the brain block assembly process can result in sections being taken from different depths.

### Potential solution

Use a razor to separate the 2 brain halves from each other. Reapply OCT to the merging sides of the brains and attempt realignment. Repeat as necessary until alignment is achieved.

## Resource availability

### Lead contact

Further information and requests for resources and reagents should be directed to and will be fulfilled by the lead contact, Ken Nakamura (ken.nakamura@gladstone.ucsf.edu).

### Materials availability

This study did not generate any new unique reagents.

## Data Availability

This study did not generate or analyze new datasets or code.
